# P-686. Evolving Patterns Of Testing For Respiratory Syncytial Virus In The United States: Who Is Getting Tested?

**DOI:** 10.1093/ofid/ofaf695.899

**Published:** 2026-01-11

**Authors:** Paul McDwyer, Harriet Dickinson, Wenyi Wang, Rikisha Gupta, Anand Chokkalingam

**Affiliations:** Gilead Sciences, Dublin, Dublin, Ireland; Gilead Sciences, Dublin, Dublin, Ireland; Gilead Sciences, Inc. Foster City, CA, USA, Foster City, California; Gilead Sciences, Inc., Foster City, CA; Gilead, Foster City, California

## Abstract

**Background:**

Patterns of Respiratory Syncytial Virus (RSV) testing have evolved over time. This study analysed how RSV testing patterns changed during 2017-2023 in a commercially insured population within the US.

**Methods:**

Patient data from 01/09/2017 to 31/08/2023 was extracted from Optum Clinformatics® Data Mart Database (CDM). The season was defined as September to August. Patients were grouped by age at the start of the season under study each year: 0-5, 18+ (adults), and 65+ years (older adults). Patients with > 6 months healthcare coverage were included in the analysis. The RSV test type, location, and result were descriptively assessed.

**Results:**

From 2017-2023 the RSV testing rate increased across all age groups: 732.9% in the 0-5 age group, 270.5% in adults, and 206.5% in older adults. The COVID-19 pandemic briefly changed RSV testing behaviour for adults: the number of tests in 2020-2021 compared to 2019-2020 reduced by 78.7% in adults and reduced even further by 81.3% in older adults. However, this reduction in testing was not seen in the in the 0-5 age group, which had a 15.3% increase.

Test type and location was assessed. From 2017-2023 there was a 123.0% increase in the proportion of tests classed as panel tests in the 0-5 age group, but a decrease of 20.4% in adults and decrease of 31.4% in older adults. Where testing location could be extrapolated, tests were requested in office/clinic settings for 38.1% of 0-5 tests (n=612) but only for 10.1% of adult tests (n=2,119). In contrast, tests were requested in acute inpatient care for 46.5% of adult tests, but only for 12.3% of 0-5 tests.

During 2022-2023, a higher proportion of RSV tests conducted were classed as RNA tests in adult (80.9%) and older adult populations (81.7%), compared to the 0-5 age group (51.8%). Additionally, during this year test positivity was higher in adult (10.1%) and older adult populations (10.8%), compared to the 0-5 age group (3.5%).

**Conclusion:**

Our research revealed a substantial increase in RSV testing from 2017 to 2023, with a large decrease during the COVID-19 pandemic. Testing approaches appear to differ between older and younger populations. These trends could be driven by novel vaccination options, greater RSV awareness and the need to distinguish between RSV and COVID-19 infection.

**Disclosures:**

Paul McDwyer, Masters, Gilead Sciences: employment|Gilead Sciences: Stocks/Bonds (Public Company) Harriet Dickinson, PhD, Gilead Sciences: Employment|Gilead Sciences: Stocks/Bonds (Public Company) Wenyi Wang, MS, Gilead Sciences: Employee and shareholder Rikisha Gupta, MPH, Gilead Sciences: Employment|Gilead Sciences: Stocks/Bonds (Public Company) Anand Chokkalingam, PhD, Gilead Sciences: Employment|Gilead Sciences: Stocks/Bonds (Public Company)

